# Effect of imatinib on plasma glucose concentration in subjects with chronic myeloid leukemia and gastrointestinal stromal tumor

**DOI:** 10.1186/s12902-018-0303-x

**Published:** 2018-11-03

**Authors:** Miguel Ángel Gómez-Sámano, Jorge Enrique Baquerizo-Burgos, Melissa Fabiola Coronel Coronel, Buileng Daniela Wong-Campoverde, Fernando Villanueva-Martinez, Diego Molina-Botello, Jose Alonso Avila-Rojo, Lucía Palacios-Báez, Daniel Cuevas-Ramos, Francisco Javier Gomez-Perez, Alejandro Zentella-Dehesa, Álvaro Aguayo-González, Alfonso Gulias-Herrero

**Affiliations:** 1 Department of Endocrinology and Metabolism, Instituto Nacional de Ciencias Medicas y Nutricion Salvador Zubiran, Vasco de Quiroga #15, Sección XVI Tlalpan, 14000 Mexico City, Mexico; 2 Universidad Catolica de Santiago de Guayaquil, Av. Carlos Julio Arosemena Km. 1½ vía Daule, Guayaquil, Ecuador; 3 Department of Internal Medicine, Hospital San Angel Inn, Av Chapultepec 489, Juárez, 06600 Mexico City, Mexico; 4 Universidad Anahuac Mexico Sur, Av. de las Torres No. 131, Alvaro Obregon, Olivar de los padres, 01780 Mexico City, Mexico; 5 Universidad Autonoma de Baja California, Campus Mexicali, Av. Alvaro Obregon y Julian Carrillo S/N, Colonia Nueva, 21100 Mexicali, B.C Mexico; 6 Department of Biochemistry, Instituto Nacional de Ciencias Medicas y Nutricion Salvador Zubiran, Vasco de Quiroga #15, Sección XVI Tlalpan, 14000 Mexico City, Mexico; 7 Department of Hematology, Instituto Nacional de Ciencias Medicas y Nutricion Salvador Zubiran, Vasco de Quiroga #15, Sección XVI Tlalpan, 14000 Mexico City, Mexico; 8 Department of Internal Medicine, Instituto Nacional de Ciencias Medicas y Nutricion Salvador Zubiran, Vasco de Quiroga #15, Sección XVI Tlalpan, 14000 Mexico City, Mexico

**Keywords:** Imatinib, Fasting plasma glucose concentrations, Chronic myeloid leukemia, Gastrointestinal stromal tumor, Type 2 diabetes mellitus

## Abstract

**Background:**

Type 2 diabetes mellitus has become one of the most important public health concerns worldwide. Due to its high prevalence and morbidity, there is an avid necessity to find new therapies that slow the progression and promote the regression of the disease. Imatinib mesylate is a tyrosine kinase inhibitor that binds to the Abelson tyrosine kinase and related proteins. It enhances β-cell survival in response to toxins and pro-inflammatory cytokine. The aim of this study is to evaluate the effect of imatinib on fasting plasma glucose in subjects with normal fasting glucose, subjects with impaired fasting glucose and in subjects with type 2 diabetes mellitus.

**Methods:**

We identified 284 subjects diagnosed with chronic myeloid leukemia or gastrointestinal stromal tumors from the Instituto Nacional de Ciencias Medicas y Nutricion Salvador Zubiran database. 106/284 subjects were treated with imatinib. We compared the effect of imatinib on fasting plasma glucose after 1 and 6 months of treatment. We used ANOVA test of repeated samples to determine statistical significance in fasting plasma glucose before imatinib treatment and the follow-up. Statistical analysis was performed with Statistical Package for the Social Sciences v22.

**Results:**

We included a total of 106 subjects: 76 with fasting plasma glucose concentrations < 100 mg/dL (normal FG), 19 subjects with fasting plasma glucose concentrations ≥100 mg/dL (impaired fasting glucose), and 11 subjects with ≥126 mg/dL (type 2 diabetes mellitus). We found a significant increase in fasting plasma glucose concentration in the normal fasting glucose group (*p* = 0.048), and a significant decrease in fasting plasma glucose concentration in the type 2 diabetes mellitus group (*p* = 0.042). In the impaired fasting glucose group, we also found a tendency towards a decrease in fasting plasma glucose (*p* = 0.076). We identified 11 subjects with type 2 diabetes mellitus, of whom, 7 (64%) had a reduction in their fasting plasma glucose concentrations after 6 months. A significant glycosylated hemoglobin reduction (*p* = 0.04) was observed.

**Conclusion:**

Subjects with chronic myeloid leukemia or gastrointestinal stromal tumor with type 2 diabetes mellitus had a significant reduction in fasting plasma glucose and glycosylated hemoglobin at 1 and 6 months while using imatinib.

**Electronic supplementary material:**

The online version of this article (10.1186/s12902-018-0303-x) contains supplementary material, which is available to authorized users.

## Background

Type 2 diabetes mellitus (T2DM) has become one of the most important public health concerns worldwide, reaching epidemic proportions. Currently, T2DM affects over 425 million people and is estimated that the number of cases will reach 629 million by 2045 [1]. Due to its high prevalence and morbidity, there is a necessity to find new therapies that slow the progression and promote the regression of the disease. Previous publications have shown an improvement of glucose metabolism with the use of imatinib in diabetic subjects with chronic myeloid leukemia (CML) and gastrointestinal stromal tumors (GIST) [2, 3].

Imatinib mesylate is a tyrosine kinase inhibitor (TKI) that binds to the Abelson tyrosine kinase (c-Abl) and related proteins. It became the very first molecular inhibitor drug to be clinically approved [4]. Also, it inhibits the platelet-derived growth factor receptor (PDGFR) and transmembrane stem-cell factor receptor (c-Kit) [5, 6], as well as it enhances β-cell survival in response to toxins and pro-inflammatory cytokines [7, 8].

The aim of this study is to evaluate the effect of imatinib on fasting plasma glucose (FPG) concentrations in subjects with CML and GIST, classified by their FPG status: normal fasting glucose (normal FG) compared to subjects with impaired fasting glucose (IFG) and subjects with T2DM. Our hypothesis is that subjects with IFG or T2DM will show an improvement in fasting glucose concentrations.

## Methods

This is a retrospective cohort study that included subjects with a diagnosis of CML or GIST that received treatment with imatinib from January 2000 to October 2016. The study was submitted and approved by the Instituto Nacional de Ciencias Medicas y Nutricion Salvador Zubiran Comite de etica en Investigacion/Comite de investigacion on July 11th, 2016.

### Study population

**Fig. 1:**
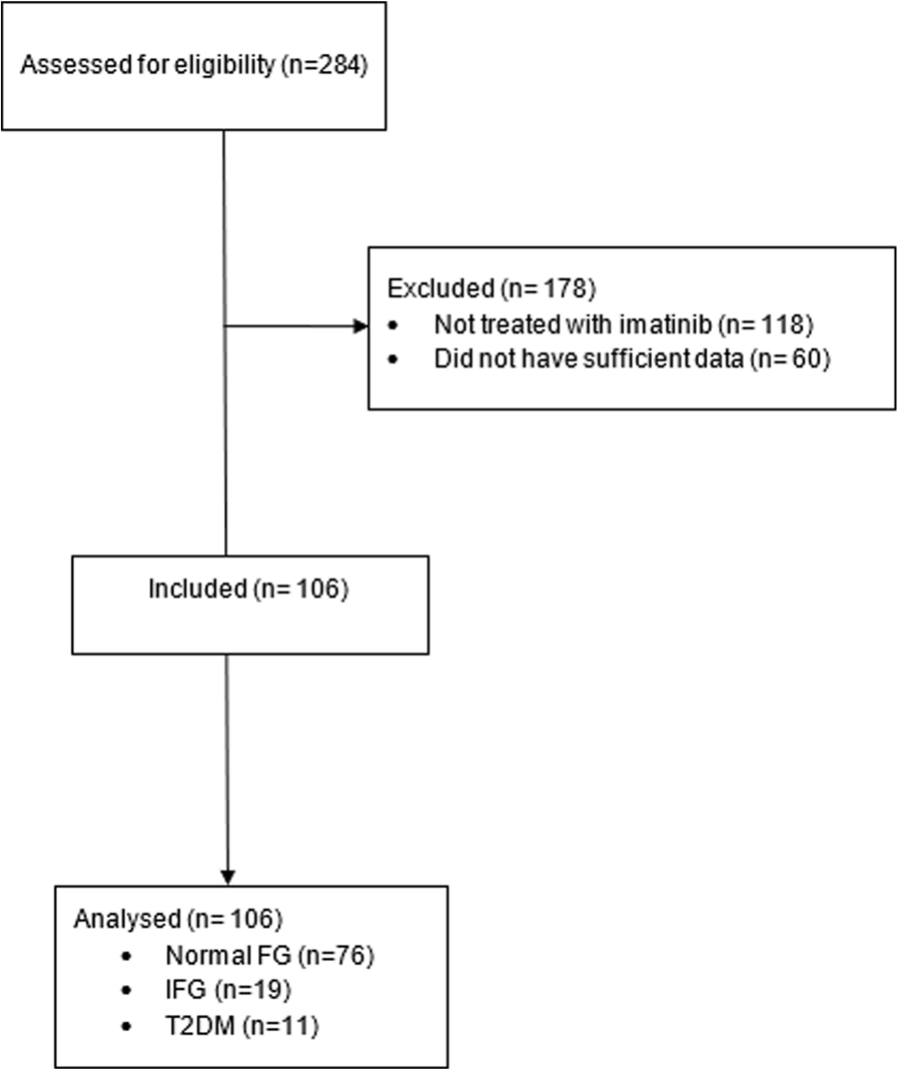
Flow chart. Normal fasting glucose; Normal FG, impaired fasting glucose; IFG, Type 2 Diabetes Mellitus; T2DM

We identified 284 Hispanic subjects diagnosed with CML and GIST from the Instituto Nacional de Ciencias Medicas y Nutricion Salvador Zubiran (INCMNSZ) database. Of these, 106 were treated with imatinib. We selected subjects aged 18 years and older. We excluded subjects who did not have enough data, such as 1-month follow-up, FPG, triglycerides (Tg), low-density lipoprotein (LDL), high-density lipoprotein (HDL), and subjects treated with imatinib in another hospital (Fig. 1).

Three study groups were formed depending on their FPG concentrations: subjects with FPG < 100 mg/dL, subjects with FPG ≥100 to < 126 mg/dL (IFG), and FPG ≥ 126 mg/dL (T2DM). Data were collected following the next criteria:

- Basal information was collected with a maximum period of 3 months prior to the treatment with imatinib.
- 1-month follow-up: information was collected with a period of ±2 weeks of completing 1 month of treatment with imatinib.
- 6-month follow-up: information was collected with a period of ±1 month of completing 6 months of treatment with imatinib.

### Biochemical and anthropometric measurements

We reviewed subject charts to retrieve laboratory data from the central laboratory of INCMNSZ, including fasting glucose, HbA1c, total cholesterol, LDL, HDL, triglycerides (Tg), alanine aminotransferase (ALT), magnesium (Mg), and creatinine. The measurements were carried out with commercially available standardized methods. In addition, other variables obtained from the subjects were age, sex, weight, and treatment for diabetes or other conditions.

### Statistical analysis

Normally distributed data are expressed as the mean and standard deviation (±SD), whereas variables with a skewed distribution are reported as median (interquartile range). In addition, we used the Kruskal-Wallis test to determine the statistical significance between the FPG concentrations before treatment and at 1 and 6 months follow-up among the three study groups. Mauchly’s sphericity test was used to evaluate equality and homogeneity of the studied population, and Greenhouse-Geisser test to obtain the *p*-value to determine if the changes of FPG were statistically and clinically significant. Data were analyzed with Statistical Package for the Social Sciences (SPSS) v22.

## Results

**Fig. 2:**
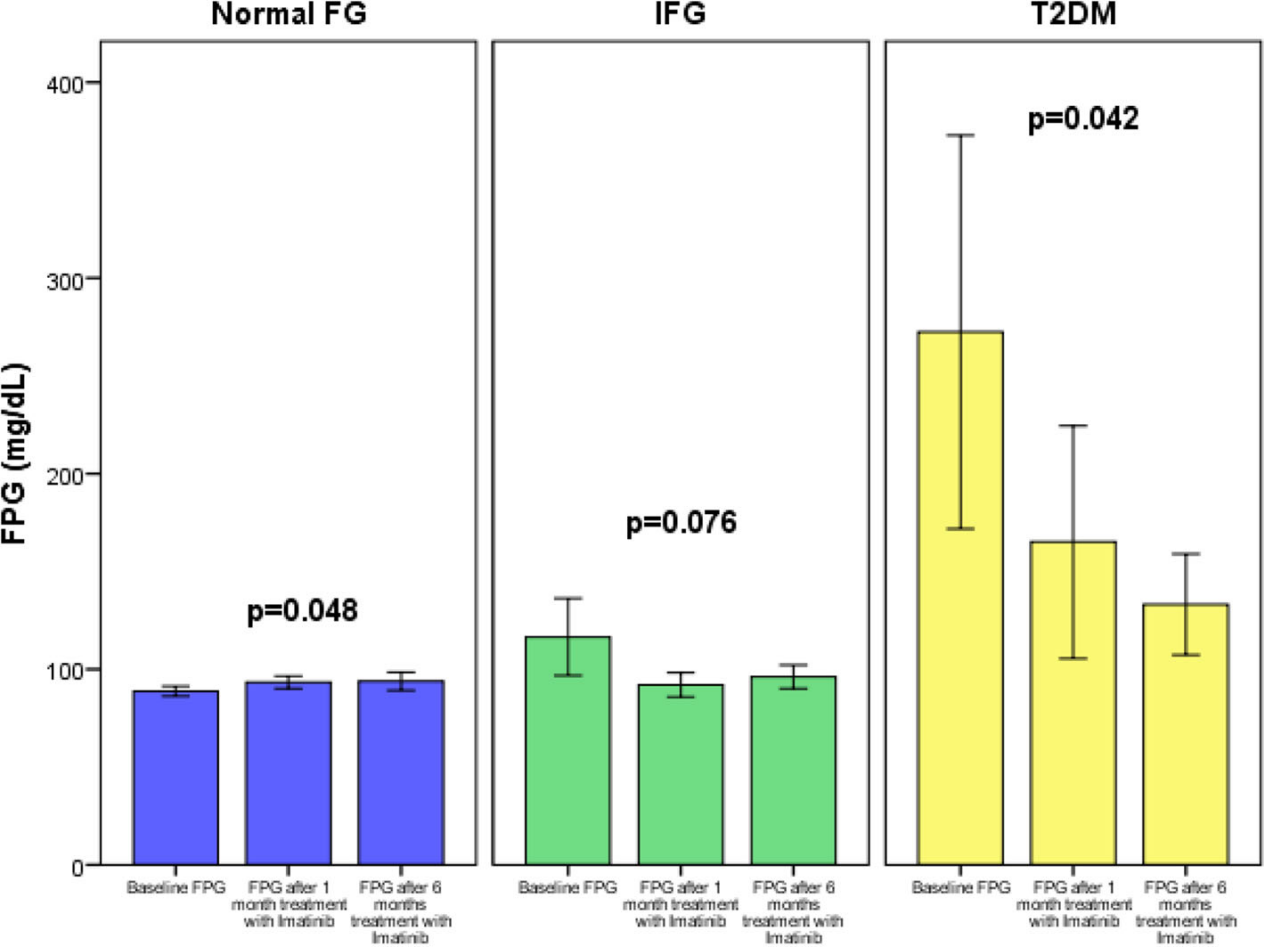
Change in fasting plasma glucose concentration after treatment with imatinib in subjects with normal fasting glucose, impaired fasting glucose and type 2 diabetes mellitus

We included a total of 106 subjects that received imatinib for 6 months: 76 with normal fasting glucose concentrations < 100 mg/dL (normal FG), 19 subjects with glucose concentrations ≥100 to < 126 mg/dL (IFG), and 11 subjects with glucose concentrations ≥126 mg/dL (T2DM). Baseline characteristics of the studied subjects are shown in Table 1. Characteristics of subjects according to their FPG concentrations are shown in Table 2, there were significant differences in age (*p* = 0.023) and ALT concentration after 1 month of treatment with Imatinib (*p* = 0.008). The comparison of mean FPG concentration variations through time in each group are presented in Table 3. We analyzed the sphericity of the mean FPG within each group with the Mauchly’s sphericity test and found a *p* = 0.960 in the normal FG group, a *p* = 0.184 in the IFG group, and a *p* = 0.702 in the T2DM group. Due to these values, we corrected the significance with the Greenhouse-Geisser test. We found a significant increase in FPG concentration in the normal FG group, before (87.6 ± 8.3), after 1 month (93.6 ± 7.6), and after 6 months (93.4 ± 10.5), *p* = 0.048; and a statistically significant decrease in FPG concentration in the T2DM group, before (241 ± 120.7), after 1 month (152.7 ± 74.5), and after 6 months (128.6 ± 33.9), *p* = 0.042. In the IFG group, there is also a decrease in FPG concentration, at baseline (121 ± 28.7), after 1 month (97. 3 ± 12.8), and after 6 months (96. 1 ± 7.3), *p* = 0.076. The latter result shows imatinib’s tendency to decrease FPG concentration, even though is not statistically significant. These results are represented in Fig. 2, where we can observe an important reduction of FPG concentration in the group with T2DM and IFG; having a decrease in concentrations at one-month follow-up, and 6-month follow-up. On the other hand, we can observe that the normal FG group had a small, yet significant increase in the FPG concentrations. Also, we decided to compare all patients with normal fasting glucose against IFG plus T2DM, in the first group we obtained a *p* = 0.059 and in the second group a significant *p* value = 0.034. These results are shown in Additional file 1: Table S1 and Additional file 2: Figure S1.

**Table 1:** Baseline characteristics of the studied subjects (*n* = 106)

Variable	Value
Sex (male: %)	52.5
CML (%)	81.8
GIST (%)	18.2
Age (years)	40.2 ± 16.7
Weight (kg)	68.3 ± 14.4
Overweight (%)	47.7
IFG (%)	17.9
DM2 (%)	10.4
Creatinine (mg/dL)	0.93 (0.68–1.0)
ALT (U/L)	28.3 (14.2–31.0)
Mg (mg/dL)	2.1 ± 0.26
Glucose (mg/dL)	109 (86–104.5)

Variables with normal distribution are expressed as mean ± s.d. Variables with non-parametric distribution are expressed as median (interquartile range). *CML* chronic myeloid leukemia, *GIST* gastrointestinal stromal tumor, *IFG* impaired fasting glucose, *ALT* alanine aminotransferase, *Mg* magnesium

**Table 2:** Characteristics of subjects according to fasting glucose

Variable	Subjects with fasting glucose < 100 mg/dL (*n* = 76)	Subjects with fasting glucose ≥100 mg/dL and < 126 mg/dL (*n* = 19)	Subjects with T2DM (*n* = 11)	*p*
Sex (male: n, %)	41, 53.9	9, 47.4	5, 45.5	0.721
Cigarette (yes: n, %)	22, 28.9	4, 26.7	3, 30	0.663
CML (n, %)	59, 80.8	13, 72.2	10, 90.9	0.747
GIST (n, %)	14, 18.4	5, 27.8	1, 9.1	
Age (years)	38.6 ± 15.7	41.8 ± 16.8	53 ± 14.2	0.023
Weight (kg)	70.5 ± 15.1	64.2 ± 14.4	65.3 ± 10.7	0.486
Weight (after 1 month, kg)	69.6 ± 14.9	63.7 ± 12.6	74.4 ± 27.5	0.645
Weight (after 6 months, kg)	74.9 ± 16.6	78.5 ± 10	61.6 ± 7.5	0.127
Mg (mg/dL)	2.2 ± 0.25	2 ± 0.20	2.1 ± 0.38	0.098
Mg (after 1 month, mg/dL)	2 ± 0.6	1.9 ± 0.18	2.25 ± 0.21	0.343
Creatinine (before, mg/dL)	0.90 (0.68–1)	1.02 (0.62–1.13)	1.02 (0.75–1.12)	0.260
Creatinine (after 1 month, mg/dL)	0.81 (0.70–0.90)	0.94 (0.68–1.05)	0.90 (0.60–1.10)	0.549
Creatinine (after 6 months, mg/dL)	0.86 (0.73–0.99)	0.84 (0.58–1.06)	0.84 (0.73–0.98)	0.239
ALT (before, U/L)	23.6 (14–26.5)	45.8 (14.2–48.2)	31 (17–48.5)	0.293
ALT (after 1 month, U/L)	24.9 (15–30)	51.3 (19–54.7)	17.8 (13–23.5)	0.008
ALT (after 6 months, U/L)	22.3 (17–26)	23.4 (20–27.5)	16. 5 (15.2–17.7)	0.106

*p* value was obtained with Kruskal-Wallis or chi-square test

*CML* chronic myeloid leukemia, *GIST* gastrointestinal stromal tumor, *IFG* impaired fasting glucose, *Mg* magnesium, *ALT* alanine aminotransferase

**Table 3:** Repeated measures ANOVA

	Glucose before	Glucose after 1 month	Glucose after 6 months	Mauchly’s sphericity test	Greenhouse-Geisser *p* value
Subjects with fasting glucose < 100 mg/dL (*n* = 76)	87.6 ± 8.3	93.6 ± 7.6	93.4 ± 10.5	0.960	0.048
Subjects with fasting glucose ≥100 mg/dL (*n* = 19)	121 ± 28.7	97. 3 ± 12.8	96. 1 ± 7.3	0.184	0.076
Subjects with T2DM (*n* = 11)	241 ± 120.7	152.5 ± 74.5	128.6 ± 33.9	0.702	0.042

Database was analyzed through the ANOVA test of repeated samples to determine the statistical significance between normal FG, IFG and T2DM subjects before treatment and at 1 and 6 months follow-up. The Mauchly’s sphericity test was used to evaluate equality and homogeneity of the studied population, and the *p* value was obtained through the Greenhouse-Geisser test

We identified 11 subjects with T2DM, which are represented in Table 4, from the INCMNSZ database. As shown in the table, two subjects (18.2%) had an increase in FPG concentration after 1 month of treatment with imatinib, one did not have sufficient follow-up data, and the remaining (72.7%) decreased their FPG concentration. According to the FPG concentration after 6 months of treatment, one subject (9.1%) had an elevation, seven decreased their glucose concentrations (63.6%), and three did not have sufficient data. HbA1c decreased from 7.48 (±1.82) to 6.2 (±0.57), with a significant reduction at 6 months after imatinib treatment (*p* = 0.04). Finally, according to the hypoglycemic treatment, four subjects (36.4%) stayed with the same treatment, two (18.2%) reduced the doses, one (9.1%) stopped taking metformin, one (9.1%) increased doses, another one (9.1%) started using insulin, and the last two subjects did not have sufficient follow-up data after 6 months of imatinib administration. With these results, we can observe that most subjects did have a reduction in the FPG concentration at 1-month follow-up (72.7%) and 6-month follow-up (63.6%), indicating a benefit of imatinib on glucose metabolism. In Table 5 we depict the studies assessing the effect of imatinib on glucose metabolism in subjects with T2DM.

**Table 4:** Subjects with type 2 diabetes

Subjects	1	2	3	4	5	6	7	8	9	10	11
Baseline Glucose	117	173	323	321	113	110	254	261	137	386	457
Glucose after 1 month	51	187	137	135	133	106	–	186	118	334	138
Glucose after 6 months	–	164	167	158	–	107	97	–	143	116	77
HbA1c before	8.6	10	8.8	14.6	6	6.5	12.1	–	6.3	8.6	–
HbA1c after 1 month	–	–	5.2	7.5	–	–	–	11	6	6.7	–
HbA1c after 6 months	–	7.1	6.2	7.6	–	6.3	6	–	5.7	7.6	5.7
Treatment before	Glyburide 5 mg TID Metformin 500 mg TID	Metformin 500 mg TID Glyburide 5–5 – 2.5	Metformin / glyburide 500 mg/5 mg TID	Glyburide 5 mg BID Metformin 850 mg QD	Metformin 500 mg QD	Metformin 500 mg QD	Glyburide 5 mg TID Metformin 850 TID	Insulin Glargine 30 U QD Metformin 850 mg QD	Metformin / glyburide 500 mg/5 mg half dose TID	NPH 45–0-20 Lispro 10–10-2	Glyburide 5 mg TID Metformin 850 mg TID
Treatment after 6 months	Stayed the same	Metformin 500 mg TID Glyburide 5 mg TID	Metformin /glyburide 500 mg/5 mg BID	Stayed the same	–	Stayed the same	NPH insulin 25 U QD Metformin 850 mg TID	–	Stayed the same	NPH 25–0-20 Lispro 10–10-3	Suspended Metformin Glyburide 5 mg TID

*HbA1c* Glycated hemoglobin, *TID* three times a day, *BID* two times a day, *QD* once a day, *NPH* neutral protamine Hagedorn

**Table 5:** Studies assessing effect of imatinib on glucose metabolism of subjects with T2DM

Author	Year	N	Effect	Reference
Agostino et al.	2011	17	47% of the subjects could discontinue their medications. All the subjects had a reduction in FPG.	[2]
Breccia et al.	2004	7	85.7% of the subjects reduced FPG concentration allowing dose decrease of oral hypoglycemic agents.	[3]
Iurlo et al.^a^	2015	27	Improve FPG levels and reduction of the dosage of antihyperglycemic drugs.	[22]
Dingli et al. ^b^	2007	7	There was no reduction of FG, HbA1c or antidiabetic treatment in any of the patients.	[9]

*FPG* fasting plasma glucose

^a^In this study, the authors did not separate subjects with IFG and T2DM

^b^Two patients of the group with normal fasting glucose developed diabetes during the treatment with imatinib

## Discussion

We observed that imatinib reduced the FPG and HbA1c concentration in subjects with T2DM. However, due to the design of our study, we cannot establish a real therapeutic effect of imatinib in subjects with T2DM. As described previously, only three of the eleven subjects with T2DM reduced their hypoglycemic therapy, but four remained with the same anti-diabetic treatment. HbA1c reduction indicates that FG was reduced, especially in subjects with high-starting HbA1c values; as in most diabetes clinical trials the magnitude of improvement in HbA1c is related to baseline A1c, the higher the A1c the greater the drop, so when A1c is normal you cannot expect a lot of improvement.

Imatinib inhibits the phosphorylation of proteins which may result in better signaling, better function of effectors, or both, with improvement in insulin sensitivity; thus, decreasing HbA1c levels in patients with high-starting values. Therefore, we can assume there is a therapeutic benefit in patients with T2DM. We cannot attribute this effect entirely to the imatinib treatment, but data in our study suggests there is a relationship between its administration and the lowering of FPG concentration.

Due to our study population, formed by Hispanic subjects with cancer, many confounding factors can alter the results. Nausea, vomiting, weight loss, hydroxyurea, and redistribution of adipose tissue [9] could influence glucose metabolism and insulin resistance. Even though there are animal trials that confirm the effect of imatinib on insulin resistance and glucose metabolism, these confounding variables need to be evaluated in prospective studies. We could not assess the effect after discontinuing therapy as Agostino et al. [2].

Ethnicity could affect imatinib’s treatment response. Several studies compared the difference of imatinib therapy response between different ethnic groups, but few studies have enough Hispanic subjects to compare against other ethnic groups. Lee et al. performed a study with more Hispanic subjects with CML compared to non-Hispanics [60.9% vs 39.1%, respectively], and concluded that Hispanic subjects achieved better treatment responses to imatinib when compared to non-Hispanic subjects [10].

We reviewed the pathophysiology of T2DM and several animal and human studies that aimed to establish the mechanism by which imatinib lowers FPG concentration. T2DM derives from the abnormal metabolism of carbohydrates, fats, and proteins which leads to hyperglycemia and hyperlipidemia. Within time, high levels of glucose and lipids induce changes in the metabolic pathways of insulin causing impaired insulin secretion from the β-cells of pancreatic islets, insulin resistance and decreased glucose use in peripheral tissues, and abnormal hepatic glucose production. Imatinib has shown to interfere in these pathways [11].

Mice models with type 1 diabetes mellitus (T1DM) and T2DM treated with TKIs have shown beneficial effects, improving several aspects of the disease. A study by Louvet et al. reported non-obese diabetic mice with new-onset T1DM experienced the regression of the disease when treated with imatinib [7]. Additionally, Chang Qing et al. established that there is an increase in the production of insulin in residual β-cells, with or without glucose stimulation, through an indirect control of the genetic expression of insulin in response to glucose, and through the promotion of the expression of glucose transporter-2 (GLUT-2) in β-cells [12]. Furthermore, it has been proven that TKIs prevent β-cell apoptosis via activation of antiapoptotic nuclear factor kappa-light-chain-enhancer of activated B cells (NF-Kb) and/or inhibition of the proapoptotic mitogen-activated protein kinase/c-jun N-terminal kinase (MAPK/JNK) [13, 14]. Moreover, Wijesekara et al. found that adiponectin promotes the phosphorylation of the protein kinase B (Akt/pkB) and the extracellular signal-regulated kinase (ERK) which leads to protection against apoptosis and stimulation of gene expression and secretion in pancreatic beta cells [15]. It has been proven that adiponectin concentration rises three times in plasma after three months of treatment with imatinib [16].

The hypoglycemic effect of this drug might be due to the inhibition of the multiple tyrosine kinases, such as c-Abl [12], PDGFR, Akt/pkB [4], and the extracellular regulatory kinases ERK1 and ERK2 which are crucial to the control and signaling activity of cellular effectors in the insulin pathway [17]. Phosphorylation of ERK by imatinib could result in better signaling, better functioning of the effectors or both [18], and it could also have an antiapoptotic effect [17]. In addition, inhibition of vascular endothelial growth factor receptor 2 (VEGFR2) reduces the degree of islet cell inflammation (insulitis) [19]. Likewise, the tyrosine phosphorylation of insulin receptor and phosphorylation of Akt/pkB after insulin administration was dose-dependent [20]. It is noteworthy that c-kit inhibition is not required for the reversal of hyperglycemia [5]. Markers of endoplasmic reticulum (ER) stress, protein kinase RNA-like endoplasmic reticulum kinase (PERK), eukaryotic initiation factor 2α (eIF2α), phosphorylated tribbles homolog 3 protein (TRB3), C/EBP homologous protein (CHOP), and phosphorylated JNK, decreased with imatinib [21].

Several case reports and retrospective human studies have been published assessing the effect of imatinib on glucose metabolism. Salalori et al. reported a subject with T1DM with the translocation-ets-leukemia/platelet-derived growth factor receptor β (TEL/PDGFRβ) rearrangement mutation and symptomatic hypoglycemia that had a reduction in the insulin dosage after treatment with imatinib [22]. Breccia et al. performed a study of 7 diabetic subjects with CML treated with imatinib, 6 showed improvements in fasting glucose concentrations, allowing a dose decrease of oral hypoglycemic agents and insulin. Before starting imatinib, they had a mean glucose of 220 mg/dL, after 3 months of treatment the mean FPG concentration was 110 mg/dL and after 12 months 108 mg/dL. The subject who was resistant to imatinib had also a decrease in FPG concentrations [3]. A similar study by Agostino et al. analyzed the effect of multiple TKIs in glucose metabolism, and a subgroup of diabetic subjects (8 of 17, 47%) could discontinue their medications, including insulin in some of them. The mean FPG decreased in all individuals associated with treatment with TKI [2]. Additionally, other case series not only found beneficial effects of imatinib on FPG concentrations, but also in the lipid profile, lowering total, high-density lipoprotein (HDL), and low-density lipoprotein (LDL) cholesterol concentrations [16, 23]. It has been proven that imatinib’s effect on glucose concentrations is stable after the end of treatment in comparison with dasatinib, which also lowered glucose concentrations [23, 24]. It is important to recall that this study has the biggest number of subjects, compared with others with the same topic.

For future studies in this topic, it would be very important to adjust the effect of imatinib on glucose metabolism for important confounders such as weight changes.

## Conclusion

We conclude that subjects with CML or GIST with T2DM had a statistically and clinically reduction in mean FPG and HbA1c at 1 and 6 months of imatinib therapy. Clinicians should consider the hypoglycemic effect of this drug when treating CML or GIST subjects with T2DM, and investigations on this drug need to continue to discover its potential use in diabetes therapy.

## Additional files

Additional file 1: **Table S1.** Repeated measures ANOVA. (DOCX 13 kb)

Additional file 2: **Figure S1.** Fasting plasma glucose concentrations follow up. FPG, fasting plasma glucose; FG, fasting glucose; IFG, impaired fasting glucose; T2DM, type 2 diabetes mellitus. (JPG 20 kb)
