# Supplementary material for: Effect of imatinib on plasma glucose concentration in subjects with chronic myeloid leukemia and gastrointestinal stromal tumor
Source: BMC Endocr Disord. 2018 Nov 3;18:77. doi: 10.1186/s12902-018-0303-x (PMC6215634; doi:10.1186/s12902-018-0303-x)
Supplement: Supplementary file 1 — Table S1. Repeated measures ANOVA. (DOCX 13 kb) [file 12902_2018_303_MOESM1_ESM.docx]

| **Additional file 1: Table S1.** Repeated measures ANOVA. | | | | | |
| --- | --- | --- | --- | --- | --- |
|  | **Glucose before** | **Glucose after 1 month** | **Glucose after 6 months** | **Mauchly's sphericity test** | **Greenhouse-Geisser p value** |
| **Subjects with fasting glucose <100 mg/dL (n=77)** | 89 ± 6.3 | 93.4 ± 7.9 | 93.7 ± 11.5 | 0.958 | 0.059 |
| **Subjects with fasting glucose ≥100 mg/dL (n=28)** | 186.5 ± 104.5 | 133.8 ± 73 | 121.9 ± 30.1 | 0.851 | 0.034 |
| Database was analyzed through the ANOVA test of repeated samples to determine the statistical significance between normal FG and impaired FG plus T2DM subjects before treatment and at 1 and 6 months follow-up. The Maulchy sphericity test was used to evaluate equality and homogeneity of the studied population, and the p value was obtained through the Greenhouse-Geisser test. | | | | | |
