# Supplementary figures and images for: Effect of imatinib on plasma glucose concentration in subjects with chronic myeloid leukemia and gastrointestinal stromal tumor
Source: BMC Endocr Disord. 2018 Nov 3;18:77. doi: 10.1186/s12902-018-0303-x (PMC6215634; doi:10.1186/s12902-018-0303-x)

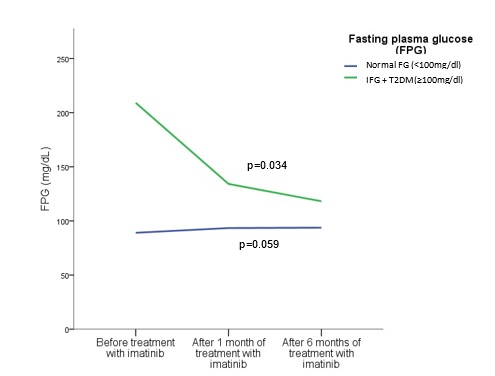

Supplement: Supplementary file 2 — Figure S1. Fasting plasma glucose concentrations follow up. FPG, fasting plasma glucose; FG, fasting glucose; IFG, impaired fasting glucose; T2DM, type 2 diabetes mellitus. (JPG 20 kb) [file 12902_2018_303_MOESM2_ESM.jpg]
